# Microarray analysis reveals an inflammatory transcriptomic signature in peripheral blood for sciatica

**DOI:** 10.1186/s12883-021-02078-y

**Published:** 2021-02-03

**Authors:** Yi Wang, Guogang Dai, Ling Jiang, Shichuan Liao, Jiao Xia

**Affiliations:** 1 Cervicodynia/Omalgia/Lumbago/Sciatica Department 2, Sichuan Provincial Orthopedics Hospital, No. 132 West First Section First Ring Road,Wuhou District, Chengdu, 610041 Sichuan Province China; 2 College Hospital, Sichuan Agricultural University-Chengdu Campus, NO. 211 Huimin Road, Wenjiang District, Chengdu, 611130 Sichuan Province China

**Keywords:** Sciatica, Peripheral blood, Differential expression, Enrichment, Network

## Abstract

**Background:**

Although the pathology of sciatica has been studied extensively, the transcriptional changes in the peripheral blood caused by sciatica have not been characterized. This study aimed to characterize the peripheral blood transcriptomic signature for sciatica.

**Methods:**

We used a microarray to identify differentially expressed genes in the peripheral blood of patients with sciatica compared with that of healthy controls, performed a functional analysis to reveal the peripheral blood transcriptomic signature for sciatica, and conducted a network analysis to identify key genes that contribute to the observed transcriptional changes. The expression levels of these key genes were assessed by qRT-PCR.

**Results:**

We found that 153 genes were differentially expressed in the peripheral blood of patients with sciatica compared with that of healthy controls, and 131 and 22 of these were upregulated and downregulated, respectively. A functional analysis revealed that these differentially expressed genes (DEGs) were strongly enriched for the inflammatory response or immunity. The network analysis revealed that a group of genes, most of which are related to the inflammatory response, played a key role in the dysregulation of these DEGs. These key genes are Toll-like receptor 4, matrix metallopeptidase 9, myeloperoxidase, cathelicidin antimicrobial peptide, resistin and Toll-like receptor 5, and a qRT-PCR analysis validated the higher transcript levels of these key genes in the peripheral blood of patients with sciatica than in that of healthy controls.

**Conclusion:**

We revealed inflammatory characteristics that serve as a peripheral blood transcriptomic signature for sciatica and identified genes that are essential for mRNA dysregulation in the peripheral blood of patients with sciatica.

**Supplementary Information:**

The online version contains supplementary material available at 10.1186/s12883-021-02078-y.

## Background

Sciatic neuralgia, which is usually referred to as “sciatica”, is a common condition involving peripheral neuropathy with a lifetime incidence of up to 40% [1]. Most sciatica is caused by lumbar disc herniation, and most studies on sciatica have focused on the lumbar discs [2]. Proteomic analyses have identified proteins associated with sciatica or disc degeneration in serum or cerebrospinal fluid, and these proteins might be involved in the pathophysiological processes of sciatica [3–5]. A transcriptomic analysis of the peripheral blood of sciatica patients has not been performed, and the transcriptomic characteristics of sciatica have not been established.

Many studies have confirmed that the inflammatory response plays a broad and important role in sciatica [6–10], and inflammatory changes might be observed in the peripheral blood. However, the transcriptional changes induced by sciatica in the peripheral blood have not been characterized. We hypothesize that sciatica causes inflammatory transcriptional features in the peripheral blood and used a microarray to investigate the gene expression characteristics in the peripheral blood of patients with sciatica with the aim of macroscopically identifying the gene expression characteristics of sciatica.

## Methods

### Peripheral blood collection and ethics statement

From April 2018 through March 2019, we enrolled 25 patients with sciatica aged 19 to 54 years with an average age of 40 years and 25 healthy volunteers aged 19 to 30 years with an average age of 23 years. The eligible patients had a complaint of sciatica, and magnetic resonance imaging confirmed single-level lumbar disc herniation at the L4/5 level or L5/S1 level leading to compression of the corresponding nerve root. Patients were excluded for other concomitant neuropathies, other spine diseases, infection, rheumatism, cardiovascular disease, metabolic disease, dementia or mental health disorders or a history of surgery, congenital disease, tuberculosis or tumour. Pregnant and lactating women were excluded. Patients with any medication record within the previous 3 months were also excluded. Peripheral blood was drawn and collected as described previously [11]. From each participant, 10 mL of fasting peripheral blood was collected from the left median cubital vein between 7:00 and 7:30 AM. All the blood samples were immediately incubated in a PAX gene Blood RNA tube (BD, USA) for less than 72 h at − 20 °C and sent to Shanghai Bohao Biotechnology Co., Ltd. (Shanghai, China) for gene chip hybridization screening. Ethical approval for this study was obtained from the Ethics Committee of the Sichuan Provincial Orthopedic Hospital. All the participants provided informed written consent.

### Microarray analysis

Chip scanning was accomplished with an Agilent Microarray Scanner platform (Agilent Technologies, Inc.). Gene chip hybridization screening was performed using an Agilent SurePrint G3 human gene expression microarray 8 × 60 K at Shanghai Biotechnology Co., Ltd., following the standard protocol established by Agilent Technologies, Inc.

### Differentially expressed genes (DEGs)

The chip scan data were log2 normalized for comparative analyses. Unrecognized probes were discarded. We deleted unrecognized probes. The average value of the data from the probes corresponding to the same gene were used for analysis. A gene with an absolute fold change (FC) of ≥1.5 was identified as a DEG, and the FC data were filtered by t-tests (*P* < 0.05). Gene expression data sets are accessible at the GEO database (http://www.ncbi.nlm.nih.gov/geo) under the number GSE150408 and GSE124272.

### Enrichment analysis and enrichment clustering

Enrichment analysis and enrichment clustering were processed using Metascape [12]. The enrichment analysis included the following ontology terms: Gene Ontology (GO) biological process (BP), GO cellular component (CC), GO molecular function (MF) and Kyoto Encyclopedia of Genes and Genomes (KEGG) Pathway. Hierarchical clustering was performed with the enriched terms. Pairs of terms with a kappa score > 0.3 were considered a cluster, and a cluster was represented by the most significant term within the cluster. A network of hierarchical clustering of enriched terms was visualized in Cytoscape software (V3.6.1).

### Protein-protein interaction (PPI) network

We used the STRING database to construct a PPI network of DEGs with a combined score > 0.4. Cytoscape software (V3.6.1) was used for visualization of the PPI network. Disconnected nodes were excluded, the centrality degree of the DEGs was calculated, and submodules were clustered, as previously described [11].

### RNA extraction and quantitative real-time quantitative PCR (qRT-PCR)

Total RNA from blood cells was extracted and purified using a PX Blood RNA Kit (Omega Bio-Tek, Inc.) following the manufacturer’s recommended protocol. Each RNA sample was reverse-transcribed into cDNA using a ReverTra Ace qPCR Kit (Toyobo), and the obtained cDNA was subsequently amplified by real-time qPCR. The gene expression levels were quantified using a 7500 HT Sequence Detection System with Power SYBR Green PCR Master Mix (both from Applied Biosystems; Thermo Fisher Scientific, Inc.). The primers for specific genes were designed with Primer Express (V3.0.1, https://www.thermofisher.com) and synthesized by Sangon Biotech Co. All forward and reverse primers used for qPCR are listed in Supplemental Table 1. The thermocycling conditions consisted of an initial denaturation at 95 °C for 10 min, 40 cycles of 95 °C for 10 s and 60 °C for 1 min, and a final extension at 60 °C for 1 min. The β-actin gene was used as an internal control, and the gene expression levels were normalized to those of β-actin according to the 2^-Δ∆Cq^ method. Significant differences in expression levels were determined using Student’s t-test with a *P*-value < 0.05. To rule out bias, the expression levels of 10% of the DEGs were validated by qRT-PCR, following the guideline recommended by Miron et al. [13].

## Results

### DEGs

**Fig. 1:**
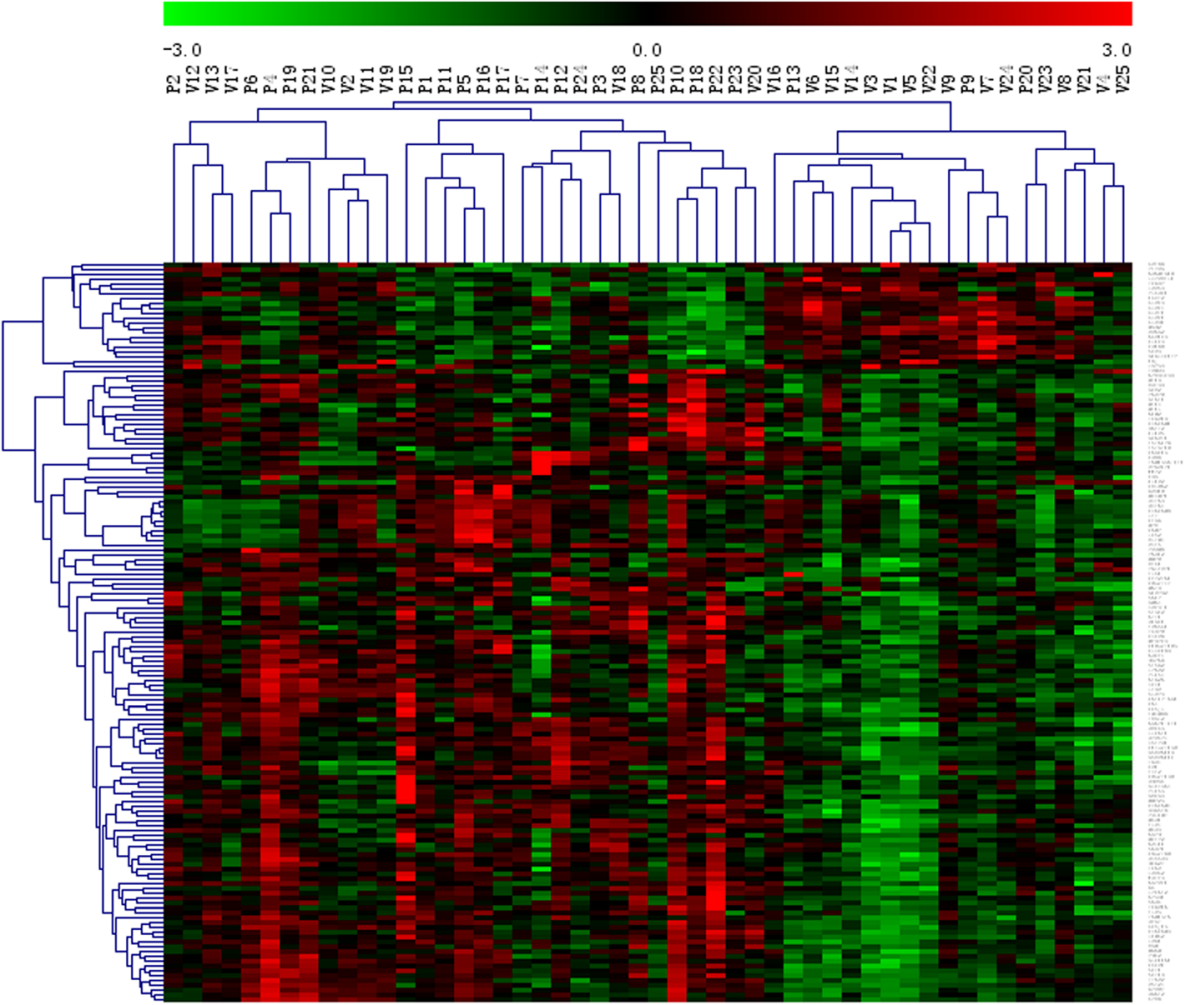
Heatmap displaying the up- and downregulation of the DEGs. The expression data were normalized using the z-score for indexes between − 3 and 3. Red, upregulated; green, downregulated. P: patient with sciatica; V: healthy volunteer

We identified 153 DEGs (Supplementary Table 2), including 131 upregulated genes and 22 downregulated genes, in the peripheral blood of the patients with sciatica compared with that of healthy controls. A heat map (Fig. 1) shows the hierarchical clustering of these DEGs.

### Enrichment analysis and enrichment clustering

**Fig. 2:**
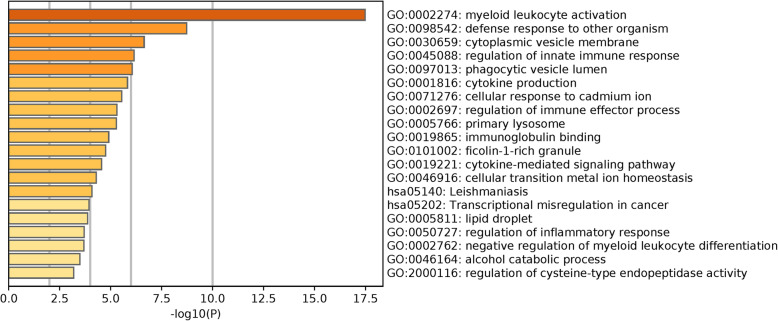
Top 20 significant clusters of the enriched terms according to the *P*-values. A cluster is represented by the most significantly enriched term within the cluster. “Log10(P)” is the *P*-value in log base 10

**Fig. 3:**
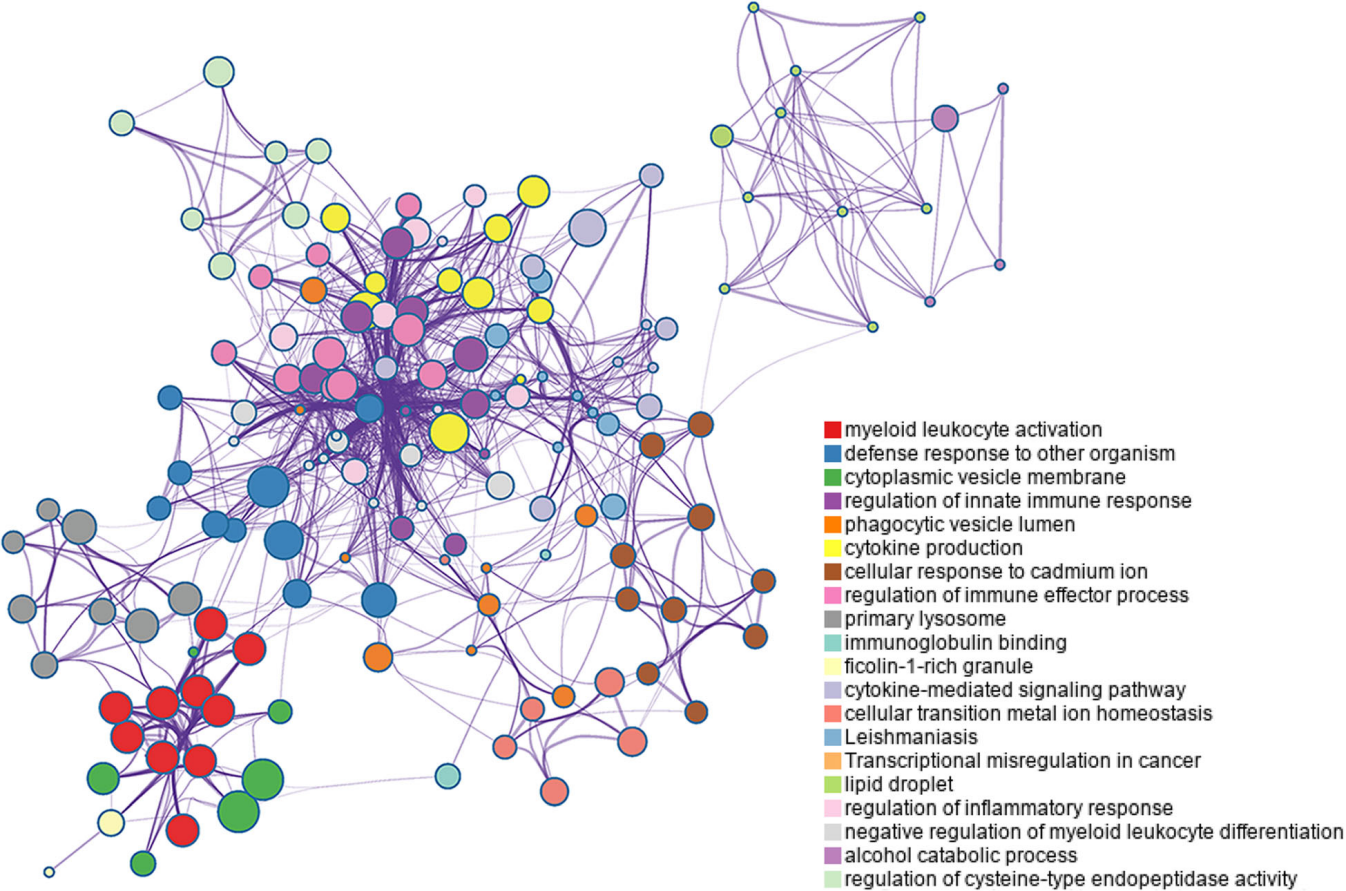
Network of the top 20 significant clusters of the enriched terms according to *P*-value. Enrichment networks were created by representing each enriched term as a node and connecting pairs of nodes with kappa scores above 0.3. Each node represents an enriched term and is coloured by cluster

Metascape analysis showed that these DEGs were enriched in 198 GO BP terms, 28 GO CC terms, five GO MF terms and 13 KEGG pathways. Notably, very strong enrichment for the BP of myeloid leukocyte activation was observed. The top 11 most significantly enriched terms were all related to the inflammatory response or immunity (Table 1). A hierarchical clustering of the enriched terms showed that 11 of the top 20 most significant clusters (Table 2 and Fig. 2) were related to the inflammatory response or immunity, and these included myeloid leukocyte activation, defence response to other organism, regulation of innate immune response, phagocytic vesicle lumen, cytokine production, regulation of immune effector process, immunoglobulin binding, phagocytic vesicle, cytokine-mediated signalling pathway, regulation of inflammatory response, and negative regulation of myeloid cell differentiation (a cluster was represented by the most significant term within the cluster). A network of the top 20 clusters of enriched terms is shown in Fig. 3.

**Table 1:** Top 11 significantly enriched terms. “Count” is the number of DGEs with membership in the given term. “Log10(P)” is the *P*-value in log base 10

GO	Term	Description	-Log10(P)	Count
GO:0002274	GO BP	myeloid leukocyte activation	17.49	30
GO:0002366	GO BP	leukocyte activation involved in immune response	16.51	30
GO:0002263	GO BP	cell activation involved in immune response	16.44	30
GO:0043312	GO BP	neutrophil degranulation	15.68	25
GO:0002283	GO BP	neutrophil activation involved in immune response	15.62	25
GO:0002275	GO BP	myeloid cell activation involved in immune response	15.50	26
GO:0042119	GO BP	neutrophil activation	15.41	25
GO:0002446	GO BP	neutrophil mediated immunity	15.40	25
GO:0036230	GO BP	granulocyte activation	15.31	25
GO:0043299	GO BP	leukocyte degranulation	14.70	25
GO:0002444	GO BP	myeloid leukocyte mediated immunity	14.37	25

**Table 2:** Top 20 significant clusters of the enriched terms according to *p* value. A cluster is represented by the most significantly enriched term within it. “Count” is the number of DGEs with membership in the given term. “%” is the percentage of DEGs that are found in the given ontology term. “Log10(P)” is the *P*-value in log base 10

GO	Category	Description	Count	%	-Log10(P)
GO:0002274	GO BP	myeloid leukocyte activation	30	20.55	17.49
GO:0098542	GO BP	defense response to other organism	19	13.01	8.73
GO:0030659	GO CC	cytoplasmic vesicle membrane	19	13.01	6.65
GO:0045088	GO BP	regulation of innate immune response	14	9.59	6.15
GO:0097013	GO CC	phagocytic vesicle lumen	3	2.05	6.06
GO:0001816	GO BP	cytokine production	18	12.33	5.83
GO:0071276	GO BP	cellular response to cadmium ion	5	3.42	5.55
GO:0002697	GO BP	regulation of immune effector process	13	8.9	5.31
GO:0005766	GO CC	primary lysosome	8	5.48	5.28
GO:0019865	GO MF	immunoglobulin binding	4	2.74	4.91
GO:0101002	GO CC	ficolin-1-rich granule	8	5.48	4.76
GO:0019221	GO BP	cytokine-mediated signaling pathway	16	10.96	4.56
GO:0046916	GO BP	cellular transition metal ion homeostasis	6	4.11	4.29
hsa05140	KEGG Pathway	Leishmaniasis	5	3.42	4.09
hsa05202	KEGG Pathway	Transcriptional misregulation in cancer icnacercancer	7	4.79	3.94
GO:0005811	GO CC	lipid droplet	5	3.42	3.87
GO:0050727	GO BP	regulation of inflammatory response	11	7.53	3.71
GO:0002762	GO BP	negative regulation of myeloid leukocyte differentiation	4	2.74	3.69
GO:0046164	GO BP	alcohol catabolic process	4	2.74	3.49
GO:2000116	GO BP	regulation of cysteine-type endopeptidase deendopeptidase endopeptidase activity endopeptidase activityendopeptidase activityendopeptidase activity	7	4.79	3.19

### PPI network of DEGs and module analysis

**Fig. 4:**
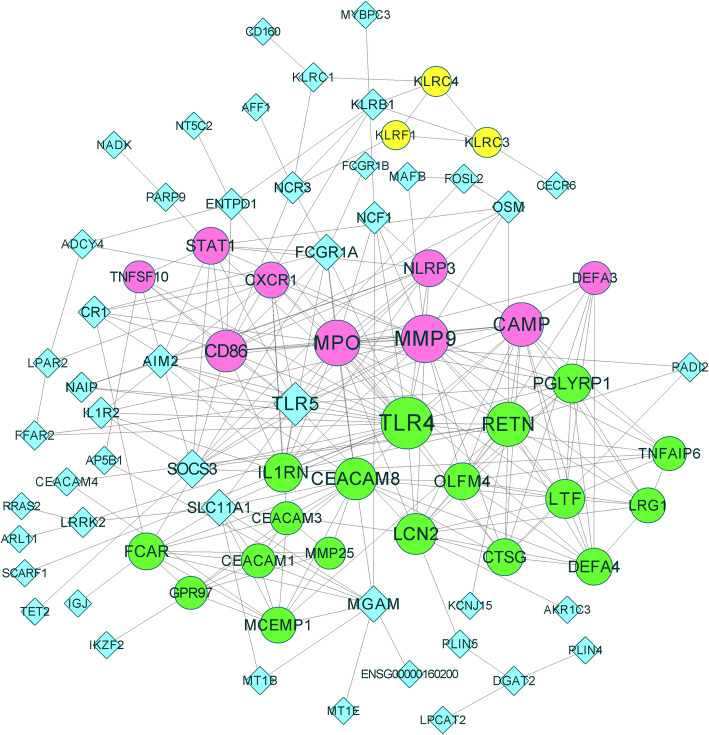
PPI net network of DEGs. Seventy-seven connected nodes and 255 edges were included. The size of the nodes represents the centrality degree. MCODE analysis identified three clusters: cluster 1 in green, cluster 2 in fuchsia and cluster 3 in yellow

The PPI network of DEGs consisted of 77 connected nodes and 255 edges (Fig. 4). To identify the key genes in the PPI network, we used the plug-in CentiScaPe to calculate the centrality degree of each node. The DEGs with a high centrality degree included TLR4 (degree, 26), MMP9 (degree, 22), MPO (degree, 20), CAMP (degree, 18), RETN (degree, 18), and TLR5 (degree, 17). The top 15 genes with the highest centrality degree are listed in Table 3. The MCODE analysis identified three clusters of the PPI network (Fig. 4). TLR4 and RETN were enriched in cluster 1, whereas MMP9, MPO, and CAMP were enriched in cluster 2. We also found that TLR4, MMP9, MPO, CAMP and RETN were enriched in the top three most significant terms, whereas MMP9, MPO, CAMP and RETN were enriched in the top five most significant terms of the first cluster of enriched terms (Table 1).

**Table 3:** Top 15 nodes with the highest centrality degree. Centrality degree was calculated by plug-in CentiScaPe and the clusters were screemed out by MCODE analysis

Gene	Degree	MCODE_Cluster
TLR4	26	Cluster 1
MMP9	22	Cluster 2
MPO	20	Cluster 2
CAMP	18	Cluster 2
RETN	18	Cluster 1
TLR5	17	Unclustered
CEACAM8	16	Cluster 1
CD86	16	Cluster 2
LCN2	15	Cluster 1
IL1RN	13	Cluster 1
PGLYRP1	13	Cluster 1
LTF	13	Cluster 1
SOCS3	12	Unclustered
CTSG	11	Cluster 1
STAT1	11	Cluster 2

### Verification of gene expression by qRT-PCR

**Fig. 5:**
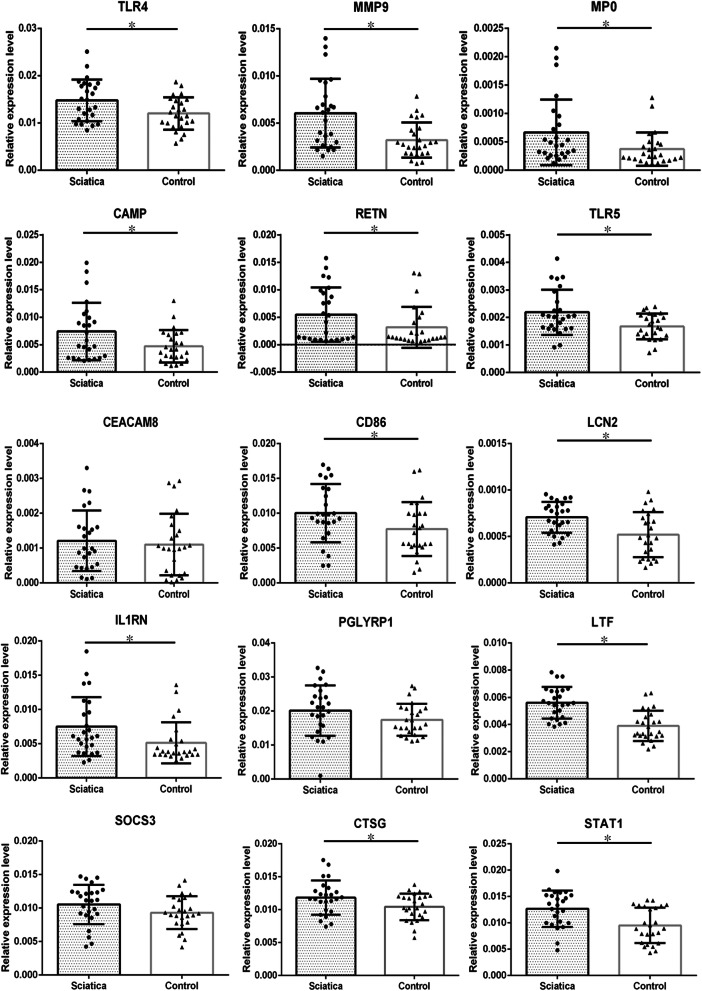
Expression of key genes. qRT-PCR results showed that the expression levels of TLR4, MMP9, MPO, CAMP, RETN and TLR5 in the peripheral blood of the patients with sciatica were significantly higher than those of the healthy controls. *: *P* < 0.05

The expression levels of top 15 genes with the highest centrality degree were validated in the peripheral blood samples using qRT-PCR. These genes included key genes identified in the above analysis. Consistent with the microarray data, the expression levels of the key genes (TLR4, MMP9, MPO, CAMP, RETN and TLR5) were significantly increased in patients with sciatica compared with healthy controls (Fig. 5).

## Discussion

We report the first analysis of gene expression in the peripheral blood for sciatica. We identified differentially expressed transcripts between patients with sciatica and healthy controls. The top 11 significantly enriched terms were all related to inflammatory response or immunity, and hierarchical clustering of enrichment revealed that more than half of the top 20 significant clusters were related to inflammatory response or immunity, indicating an inflammatory and immune characteristic in peripheral blood for sciatica. Enrichment analysis also revealed a strong enrichment for the BP of myeloid leukocyte activation. We also found that these DEGs were enriched in 13 KEGG pathways. However, there are no reports on the relationship between these terms (including myeloid leukocyte activation) and sciatica and or on the relationship between these KEGG pathways and sciatica. Our study might provide a novel understanding of the mechanism of sciatica. Further research of the genes identified in this study is needed to determine the detailed mechanism of sciatica.

Previous studies have reported serum inflammatory biomarkers of sciatica, and these include TNF-α, IL-4, IL-6, IL-8, IL-10, IL-17, IL-21, T helper lymphocytes 17, phospholipase A2, C-reactive protein, C-X3-C motif ligand 1, C-C motif ligand 2 and mast cell proteinase-1 [14–22]. Increases in the levels of these serum inflammatory factors indicate the inflammatory characteristics of the peripheral blood of patients with sciatica, which is consistent with the enrichment results obtained in our study. Enzyme-linked immunosorbent assays, mRNA/qPCR, proximal extension assays, western blotting and latex agglutination were used in these previous studies. None of these biomarkers were identified by our microarray analysis, which might be due to the different techniques and sensitivities. The main difference between our studies and existing reports is that we employed a microarray to detect all mRNAs in the peripheral blood, while other studies tested for specific molecules or genes that are already known to be potentially involved in sciatica.

Through proteomic analyses, Xie et al. [3] found that the protein levels of apolipoprotein-L1 and two serum albumin precursors were upregulated and that those of apolipoprotein M, tetranectin and immunoglobulin light chain were downregulated in patients with sciatica. Sarath et al. [4] identified 73 differentially expressed proteins in a degenerated annulus fibrosus in comparison with a normal disc, but none of the mRNAs of these proteins were identified as DEGs in our study. These researchers identified 54 differentially expressed proteins in a degenerated nucleus pulposus compared with a normal disc, and among these 54 proteins, only the mRNA of carcinoembryonic antigen-related cell adhesion molecule 4 was differentially expressed in the peripheral blood of sciatica patients compared with that of healthy controls in our study. Liu et al. [5] identified 15 proteins that are differentially expressed in the cerebrospinal fluid of patients with sciatica compared with that of normal controls. None of the mRNAs of these proteins were found to be differentially expressed in peripheral blood in our study. Obviously, the dysregulation of DEGs identified in our study was not consistent with the results of these proteomic analyses. Because many factors are involved in the process of protein translation from mRNA, mRNA expression levels are not necessarily consistent with the corresponding protein expression levels. In addition, differences in other variables, such as age, medical history, sample source, race, control, and cut-off fold change, often lead to bias in a basic clinical study. Genomics and proteomics research on sciatica is still a long way off.

The network analysis performed in the present study showed coordinated groups of genes with biological significance and identified TLR4, MMP9, MPO, CAMP, RETN and TLR5 as the key genes in the network. Most of these key genes were related to inflammatory response and were significantly enriched in the BP of myeloid leukocyte activation. In the PPI network, TLR4 had the highest centrality degree. This finding is consistent with our previous research [11], demonstrating that TLR4 may be essential for the differential expression of genes in the whole blood of patients with lumbar disc prolapse. Of all the Toll-like receptors, TLR4 is the most studied because it plays a major role in the inflammatory response [23]. TLR4 is also involved in innate neuroimmunity and neuropathy and mediates inflammatory and neuropathic pain [24]. An animal model confirmed that the expression of TLR4 in intervertebral discs and that the activation of TLR4 in intervertebral discs induces an inflammatory response, including the upregulation of TNF-α, IL-1β, IL-6 and nitric oxide [25]. The inhibition of TLR4 in discs reduces inflammation and reverses pain-related neuroplasticity, which suggests that TLR4 is a potential target for treating disc-related inflammatory and neuropathic pain [26]. The role of TLR5 in pain is unclear. TLR5-knockout mice show lower levels of pain due to nerve injury than wild-type mice, and TLR5 antagonists abrogate pain in a rat model of acute allodynia, which suggests that TLR5 is related to pain [27, 28]. MMP9 is associated with sciatica in both the peripheral blood and local discs. This molecule is known to degrade collagen and is expressed in intervertebral discs, which have high levels of collagen [29]. The MMP9 level is decreased in free, protruded, and extruded discs [30]. MMP9 is involved in the activation and inactivation of inflammation through an unknown mechanism [31]. Focal MMP9 promotes the migration of leukocytes from the peripheral region to the tissue by generating a chemotactic gradient, which indicates a role in the neuroinflammatory process [32]. MPO is mainly produced by circulating neutrophils and is linked to inflammatory conditions and degenerative neurological disorders. Though producing hypochlorous acid-sphingomyelinase, which is released as a source of reactive oxygen species, MPO indirectly mediates inflammatory injury [33]. Stroke patients present higher MPO expression in both plasma and serum [34]. The expression of MPO is increased in the brains of patients with Alzheimer’s or Parkinson’s disease [35, 36], and MPO is reportedly activated in multiple sclerosis plaques [37]. The underlying mechanism of MPO in these neurological diseases remains unclear. RETN is an adipokine with proinflammatory properties. This molecule upregulates TNF-α and IL-6 in human peripheral blood mononuclear cells as well as TNF-α and IL-12 in human macrophages [38, 39]. In many inflammatory conditions, a link between RETN levels and inflammatory markers in plasma has been shown in various studies; these markers include TNF-α receptor-2, IL-6, ICAM-1, lipoprotein-associated PLA2 and C-reactive protein [40, 41]. Although CAMP, also known as LL-37, has been studied extensively, its antibacterial activities, its roles in the immune system and cancer [42], and its connection with sciatica have not been explored.

Our study has limitations. First, we enrolled patients with sciatica secondary to lumbar disc herniation. Lumbar disc herniation is based on disc degeneration. Disc degeneration is an age-related process [43], and the degree of histopathologic degeneration in a disc increases with age [44]. All the patients with sciatica included in the present study were confirmed to exhibit disc herniation by MRI. The literature does not detail to what degree of histopathologic degeneration can disc degeneration be observed by MRI. If the two groups are age-matched, healthy volunteers are likely to present a higher degree of histopathologic degeneration than young people, and the histopathologic degeneration might not be confirmed by images, which could affect the results of a microarray analysis. On the other hand, young people are more representative of people without disc degeneration or lower disc degeneration; thus, we recruited young people as controls. Based on this grouping, DEGs might be related to other aging process. Both grouping methods have pros and cons. Second, we have performed several other similar studies [11, 45, 46], but the results of these studies and the present study were not much the same. All of our studies are cross-sectional studies, and due to the limitations of a cross-sectional study, the results are largely affected by the conditions at the time of the study. Third, many factors, such as miRNAs and lncRNAs, are involved in the process of protein translation from mRNA, and protein expression is not only determined by mRNA. We did not perform western blotting to confirm the protein levels of these key genes, and the role that these key genes play in the inflammatory response in sciatica and the mechanism of sciatica need further investigation.

## Conclusions

The present study demonstrates that 153 genes are differentially expressed in the peripheral blood of patients with sciatica. Notably, enrichment analysis revealed that most of the significantly enriched terms are related to inflammatory response or immunity. Network analysis identified TLR4, MMP9, MPO, CAMP, RETN and TLR5 as key genes contributing to the dysregulation of genes in the peripheral blood of patients with sciatica. Most of these key genes were related to inflammatory response in sciatica. In conclusion, we revealed inflammatory characteristics as a peripheral blood transcriptomic signature for sciatica and identified genes that are essential for mRNA dysregulation in the peripheral blood of patients with sciatica. Future studies should further characterize the role of the peripheral inflammatory response played in the pathology of sciatica and determine its relevance to overall disease progression.

## Supplementary Information

**Additional file 1 : Table S1.** Sequences of primers used for quantitative real-time polymerase chain reaction. F, forward; R, reverse.

**Additional file 2 : Table S2.** Differentially expressed genes in the peripheral blood between the patients with sciatica and the healthy controls. P: *P*-value; FC: fold change.

## Data Availability

The gene expression datasets generated and analysed during the current study are available in the GEO database (http://www.ncbi.nlm.nih.gov/geo) under the number GSE150408 and GSE 124272. The datasets used and/or analysed during the current study are available from the corresponding author upon reasonable request.

## Abbreviations

TNF-α: Tumour necrosis factor-α
DEGs: Differentially expressed genes
GO: Gene Ontology
BP: Biological process
CC: Cellular component
MF: Molecular function
KEGG: Kyoto Encyclopedia of Genes and Genomes
qRT-PCR: Quantitative real-time quantitative PCR
IL: Interleukin
TLR4: Toll-like receptor 4
MMP9: Matrix metallopeptidase 9
MPO: Myeloperoxidase
CAMP: Cathelicidin antimicrobial peptide
RETN: Resistin
TLR5: Toll-like receptor 5
CEACAM8: Carcinoembryonic antigen-related cell adhesion molecule 8
CD86: CD86 molecule
LCN2: Lipocalin 2
IL1RN: Interleukin 1 receptor antagonist
PGLYRP1: Peptidoglycan recognition protein 1
LTF: Lactotransferrin
SOCS3: Suppressor of cytokine signaling 3
CTSG: Cathepsin G
STAT1: Signal transducer and activator of transcription 1
