# Supplementary material for: Microarray analysis reveals an inflammatory transcriptomic signature in peripheral blood for sciatica
Source: BMC Neurol. 2021 Feb 3;21:50. doi: 10.1186/s12883-021-02078-y (PMC7856817; doi:10.1186/s12883-021-02078-y)
Supplement: Supplementary file 1 — Additional file 1 : Table S1. Sequences of primers used for quantitative real-time polymerase chain reaction. F, forward; R, reverse. [file 12883_2021_2078_MOESM1_ESM.docx]

**Supplementary Table 1.** Sequences of primers used for quantitative real-time polymerase chain reaction. F, forward; R, reverse.

| **Gene** | **Sequence (5' to 3')** |
| --- | --- |
| TLR4 | F: CCTGAGGCATTTAGGCAGCTA |
|  | R: GATAAATCCAGCACCTGCAGTTC |
| MMP9 | F: CACGCACGACGTCTTCCA |
|  | R: AAGCGGTCCTGGCAGAAAT |
| MPO | F: CGGTACCCAGTTCAGGAAGCT |
|  | R: CCCTCGTTCTCCCACCAAA |
| CAMP | F: TCAAGGATTTTTTGCGGAATCT |
|  | R: GCCAGGGTAGGGCACACA |
| RETN | F: AGCCATCAATGAGAGGATCCA |
|  | R: AGGCCAATGCTGCTTATTGC |
| TLR5 | F: TCTGCTAGGACAACGAGGATCA |
|  | R: CCATGAGCACCACTCCTAGGA |
| CEACAM8 | F: TGAGACTCAGAACACAACCTAC |
|  | R: TTCACATTCATAGGGTCCTACG |
| CD86 | F: TGCTCATCTATACACGGTTACC |
|  | R: TGCATAACACCATCATACTCGA |
| LCN2 | F: GAGTTACCCTGGATTAACGAGT |
|  | R: AAGCGGATGAAGTTCTCCTTTA |
| IL1RN | F: CAACTAGTTGCTGGATACTTGC |
|  | R: GCTCAGGTCAGTGATGTTAACT |
| PGLYRP1 | F: CACTCAGGTCACTTATGGAACC |
|  | R: GTGTCCTTTGAGCACATAGTTG |
| LTF | F: CGCCTACTGAGGTCTGAA |
|  | R: CGCCTACTGAGGTCTGAA |
| SOCS3 | F: CTTCTCTCTGCAGAGCGATC |
|  | R: ATGTAATAGGCTCTTCTGGGGG |
| CTSG | F: GGAACAGATACACTCCGAGAG |
|  | R: CTGACGACTTTCCATAGGAGAC |
| STAT1 | F: CGTCCTGAGTATTCCAAGT |
|  | R: CGTCCTGAGTATTCCAAGT |
| β-Actin | F: CTGGAACGGTGAAGGTGACA |
|  | R: CGGCCACATTGTGAACTTTG |
