# Supplementary material for: Microarray analysis reveals an inflammatory transcriptomic signature in peripheral blood for sciatica
Source: BMC Neurol. 2021 Feb 3;21:50. doi: 10.1186/s12883-021-02078-y (PMC7856817; doi:10.1186/s12883-021-02078-y)
Supplement: Supplementary file 2 — Additional file 2 : Table S2. Differentially expressed genes in the peripheral blood between the patients with sciatica and the healthy controls. P: P-value; FC: fold change. [file 12883_2021_2078_MOESM2_ESM.docx]

**Supplementary Table 2.** Differentially expressed genes in the peripheral blood between the patients with sciatica and the healthy controls. P: P-value; FC: fold change.

| **Gene** | **P** | **FC** | **Gene** | **P** | **FC** | **Gene** | **P** | **FC** |
| --- | --- | --- | --- | --- | --- | --- | --- | --- |
| PRDM8 | 0.01 | 3.6 | MCEMP1 | 0.05 | 1.6 | CEACAM3 | 0.00 | 1.5 |
| OLFM4 | 0.03 | 3.2 | C9orf47 | 0.00 | 1.6 | DHRSX | 0.00 | 1.5 |
| RPGRIP1 | 0.03 | 3.0 | LUCAT1 | 0.00 | 1.6 | CXCR1 | 0.00 | 1.5 |
| COL9A2 | 0.02 | 2.8 | NAIP | 0.01 | 1.6 | MYBPC3 | 0.00 | 1.5 |
| BATF2 | 0.02 | 2.8 | WWP2 | 0.03 | 1.6 | SCO2 | 0.04 | 1.5 |
| FCGR1A | 0.00 | 2.5 | CR1 | 0.00 | 1.6 | MXD1 | 0.00 | 1.5 |
| CEACAM8 | 0.02 | 2.4 | NCF1 | 0.00 | 1.6 | RNF24 | 0.01 | 1.5 |
| LTF | 0.00 | 2.4 | C8orf60 | 0.00 | 1.6 | ADCY4 | 0.01 | 1.5 |
| LCN2 | 0.02 | 2.2 | CATIP-AS1 | 0.00 | 1.6 | ASPH | 0.01 | 1.5 |
| DEFA4 | 0.02 | 2.2 | CEACAM4 | 0.00 | 1.6 | OSM | 0.03 | 1.5 |
| MPO | 0.00 | 2.1 | DISC1 | 0.01 | 1.6 | B3GNT8 | 0.00 | 1.5 |
| GRB10 | 0.04 | 2.1 | HCG27 | 0.00 | 1.6 | MCTP2 | 0.00 | 1.5 |
| CEACAM1 | 0.00 | 2.1 | TLR4 | 0.00 | 1.6 | ARL11 | 0.00 | 1.5 |
| CTSG | 0.02 | 2.0 | CCNJL | 0.03 | 1.6 | SNORA11C | 0.00 | 1.5 |
| MMP9 | 0.01 | 2.0 | IL1R2 | 0.05 | 1.6 | PHC2 | 0.01 | 1.5 |
| PLIN4 | 0.00 | 2.0 | MT1B | 0.04 | 1.6 | NADK | 0.01 | 1.5 |
| TNFAIP6 | 0.00 | 1.9 | CYP27A1 | 0.00 | 1.6 | AFF1 | 0.00 | 1.5 |
| DEFA3 | 0.02 | 1.9 | PARP9 | 0.02 | 1.6 | LPAR2 | 0.02 | 1.5 |
| GPR97 | 0.01 | 1.9 | DENND3 | 0.00 | 1.6 | LTBR | 0.01 | 1.5 |
| ENTPD1 | 0.00 | 1.9 | CLEC18B | 0.01 | 1.6 | FGD4 | 0.00 | 1.5 |
| PLIN5 | 0.00 | 1.9 | FFAR2 | 0.01 | 1.6 | C9orf139 | 0.00 | 1.5 |
| FCGR1B | 0.00 | 1.9 | APOBEC3B | 0.03 | 1.6 | FRMD3 | 0.04 | 1.5 |
| RETN | 0.05 | 1.9 | AP5B1 | 0.00 | 1.6 | NLRP3 | 0.00 | 1.5 |
| CASC15 | 0.00 | 1.9 | SCARF1 | 0.00 | 1.6 | TET2 | 0.00 | 1.5 |
| LRG1 | 0.00 | 1.8 | MMP25 | 0.00 | 1.6 | TRANK1 | 0.00 | 1.5 |
| MT1L | 0.04 | 1.8 | CECR6 | 0.00 | 1.6 | TLN1 | 0.02 | 1.5 |
| CAMP | 0.02 | 1.8 | LIMK2 | 0.00 | 1.6 | PADI2 | 0.03 | 1.5 |
| CBS | 0.02 | 1.8 | PDLIM7 | 0.00 | 1.6 | MAFB | 0.00 | 1.5 |
| NTNG2 | 0.01 | 1.8 | FAM155A-IT1 | 0.03 | 1.6 | ODF3B | 0.00 | 1.5 |
| SIRPB2 | 0.00 | 1.8 | TMEM88 | 0.01 | 1.6 | KLRC4 | 0.00 | 0.7 |
| DYSF | 0.00 | 1.8 | ATG2A | 0.01 | 1.6 | PTPRS | 0.00 | 0.7 |
| CA4 | 0.01 | 1.8 | SOCS3 | 0.02 | 1.6 | LRRN3 | 0.02 | 0.7 |
| DGAT2 | 0.01 | 1.7 | SNORA11B | 0.00 | 1.6 | NCR3 | 0.00 | 0.7 |
| MT1E | 0.03 | 1.7 | SLC45A4 | 0.00 | 1.6 | CLIC3 | 0.00 | 0.7 |
| FAM157A | 0.00 | 1.7 | ASPRV1 | 0.00 | 1.6 | RRAS2 | 0.00 | 0.7 |
| SLC11A1 | 0.01 | 1.7 | KCNJ15 | 0.01 | 1.6 | IKZF2 | 0.00 | 0.7 |
| ERV3-1 | 0.01 | 1.7 | XPO6 | 0.02 | 1.6 | SIGLEC17P | 0.00 | 0.7 |
| C10orf105 | 0.01 | 1.7 | TLR5 | 0.00 | 1.5 | MSH2 | 0.00 | 0.7 |
| ECE1 | 0.02 | 1.7 | STAT1 | 0.01 | 1.5 | USP53 | 0.03 | 0.6 |
| NCF1B | 0.00 | 1.7 | C14orf159 | 0.00 | 1.5 | KLRC1 | 0.00 | 0.6 |
| IL1RN | 0.01 | 1.7 | DOCK5 | 0.00 | 1.5 | PLXDC1 | 0.01 | 0.6 |
| AIM2 | 0.02 | 1.7 | NABP1 | 0.00 | 1.5 | CD160 | 0.00 | 0.6 |
| CD86 | 0.01 | 1.7 | MXD3 | 0.01 | 1.5 | KLRC3 | 0.00 | 0.6 |
| FCAR | 0.01 | 1.7 | LRRK2 | 0.00 | 1.5 | AKR1C3 | 0.00 | 0.6 |
| PGLYRP1 | 0.01 | 1.7 | NT5C2 | 0.00 | 1.5 | KLRF1 | 0.00 | 0.6 |
| FOSL2 | 0.00 | 1.7 | OTX1 | 0.02 | 1.5 | KLRB1 | 0.00 | 0.6 |
| DPRXP4 | 0.00 | 1.6 | LPCAT2 | 0.00 | 1.5 | KRT86 | 0.00 | 0.6 |
| MGAM | 0.01 | 1.6 | GK | 0.00 | 1.5 | LEPROTL1 | 0.00 | 0.6 |
| ASAP1-IT1 | 0.00 | 1.6 | SMA4 | 0.02 | 1.5 | FCGBP | 0.00 | 0.6 |
| WDFY3 | 0.00 | 1.6 | HSPA6 | 0.00 | 1.5 | ADAMTS10 | 0.01 | 0.5 |
| TNFSF10 | 0.04 | 1.6 | FKBP9 | 0.01 | 1.5 | IGJ | 0.04 | 0.5 |
